# Annual acknowledgement of manuscript reviewers

**DOI:** 10.1186/1758-5996-5-14

**Published:** 2013-03-21

**Authors:** Daniel Giannella-Neto, Marilia de Brito Gomes, Lucy Abel

**Affiliations:** 1 Post Graduation Programme in Medicine, Universidade Nove de Julho, Rua Vergueiro, 235-249 – 2º SS, São Paulo, 01345-000, Brazil

## Abstract

**Contributing reviewers:**

The Editors of Diabetology and Metabolic Syndrome would like to thank all reviewers, both external and Editorial Board Members, who have contributed to the journal since its inception, and whose valuable support continues to be essential to the success of the journal.

## 

Nader Abraham

United States

Sean Adams

United States

Adejuwon Adeneye

Nigeria

Raghu Adya

United Kingdom

Bharat B Aggarwal

United States

Navneet Agrawal

India

Abelardo Aguilera

Spain

Bo Ahrén

Sweden

Jaweed Akhtar

United States

Baris Akinci

Turkey

Eduardo Alegria

Brazil

Adnan A Ali

Canada

Ather Ali

United States

Sabrina E M Almeida

Brazil

Marlene M Alvarez

Brazil

Andion C Alves

Brazil

Kamal Amin

Egypt

Amy Anderson

United States

Christian Andersson

Sweden

Claudia R M Andrade

Brazil

Francesco Andreozzi

Italy

Theodore Angelopoulos

United States

Tiago Antunes-Lopes

Brazil

Peter Apor

Hungary

David Araújo-Vilar

Spain

Ehud Arbit

United States

Pablo Aschner

Colombia

Yoshimasa Aso

Japan

Nimer Assy

Israel

Martin Auinger

Austria

Fabienne Aujard

France

Navneet Aujla

United Kingdom

Márcio Augusto Averbeck

Brazil

Álvaro Avezum

Brazil

Daud M. Ishaq Aweis

Malaysia

Fereidoun Azizi

Iran

Mazen S Bader

Canada

Adrian Bagust

United Kingdom

Mahmoud E Balbaa

Lebanon

Sandra S M Barbalho

Brazil

Pasqual Barretti

Brazil

Emil Daniel Bartels

Denmark

Abdul Basit

Poland

John Batsis

United States

Roy W Beck

United States

Carl Johan Behre

Sweden

Lars Bellner

United States

Israel Bendit

Brazil

Isabela Bensenor

Brazil

Darlene Berryman

United States

Estela Bevilacqua

Brazil

Madhur Bhattarai

Nepal

Aurelian Bidulescu

United States

Jaap Van Der Bijl

Holand

Benjamin Bikman

United States

Einar S Bjornsson

Sweden

Agnieszka Blachnio-Zabielska

Poland

Vincent Blasco-Baque

France

David Boam

United Kingdom

Velio Bocci

Italy

Krishna Murthy Boini

India

Barbara J J Boucher

United Kingdom

Maher Boukhris

France

Pascal Bousquet

France

Susan Brain

United Kingdom

Natasa Bratina

Slovenia

Lusine Breitscheidel

Germany

Vera Bril

Canada

Henry Buchwald

United States

Roberto Burattini

Italy

Thomas Caceci

United States

Leah Cahill

United States

Lu Cai

United States

Luis Eduardo P Caliari

Brazil

Ian I C Campbell

United Kingdom

Kleber K Campos

Brazil

Luis Henrique Canani

Brazil

Zeynep Canturk

Turkey

Danielle Arisa Caranti

Brazil

Elena T Carbone

United States

David Castro-Diaz

United States

Hugo Castro-Faria-Neto

Brazil

Berrin Cetinarslan

Turkey

Subhankar Chakraborty

United States

Cory Champagne

United States

Prakash Chandra

United States

Ernst Chantelau

Germany

Natalie Chapados

Canada

Lisa Chasan-Taber

United States

Norberto Chavez-Tapia

Italy

Rima Chedid

Lebanon

Xiuping Chen

Macao

Andrea Cherrington

United States

Rakesh Chibber

United Kingdom

Ezenwaka Chidum

United States

Anil Baran Choudhury

India

Tore Christiansen

Denmark

George Chrousos

United States

Shao-Yuan Chuang

Taiwan

Timothy Church

United States

José Cipola-Neto

Brazil

Roberta Cobas

Brazil

Michael Cobble

United States

Sheri Colberg-Ochs

United States

Michele Colombo

Denmark

Jean O'Connell

Ireland

Maria Lucia C Correa-Giannella

Brazil

Ana Cortizo

Argentine

Evans Coutinho

United States

Silvia Cozzolino

Brazil

Mary P Cullinan

New Zealand

Pablo Cure

United States

Ghenadie Curocichin

Republic of Moldova

Cesare Cuspidi

Italy

Andrea J Cussons

Australia

Alberto Enrique D'Ottavio

Argentine

Lance Dalleck

New Zealand

Débora Damasceno

Brazil

Ana A R D Damaso

Brazil

Durval Damiani

Brazil

Susan Davis

Patrick G Dean

Australia

United States

Seyed Mohsen Dehghani

Iran

Melanie Van Der Klauw

Holand

Christopher A Desouza

United States

Ketan Dhatariya

United Kingdom

Gurdeep Singh Dhatt

United Arab Emirates

Vincenzo Di Marzo

Italy

Sergio A Dib

Brazil

John B Dixon

Australia

Christoph Dorn

Germany

Boris Draznin

United States

George Drummond

United States

Ravindranath Duggirala

United States

Bruce Duncan

Brazil

Simon Dunmore

United Kingdom

Pamela Dyson

United Kingdom

Hossam Ebaid

Saudi Arabia

Ken Ebihara

Japan

Kristin Eckardt

Germany

David Eide

United States

Dalia H El-Lebedy

United Kingdom

Mohsen Eledris

Saudi Arabia

John M Embil

Canada

Munechika Enjoji

Japan

Cihangir Erem

Turkey

Travis Esslinger

United States

Louise Færch

Denmark

Karla Fabiana S Melo C Fagundes

Brazil

Hossein Fakhrzadeh

Iran

Ziba Farajzadegan

Iran

Olufemi Fasanmade

Nigeria

Irene A Fawzy

Egypt

Christine Feillet-Coudray

France

Richard Feinman

United States

Ana Angelica H Fernandes

Brazil

Olga Sonia León Fernández

Cuba

Sarah De Ferranti

United States

Sandra Roberta Gouvea Ferreira

Brazil

Christoph Fiehn

Germany

Howard Fillit

United States

Francis Finucane

United Kingdom

Benedicte Fontaine-Bisson

Canada

Carol Forsblom

Finland

Zuleica B Fortes

Brazil

Milton Foss

Brazil

Maria Cristina Foss-Freitas

Brazil

Laercio Franco

Brazil

David Frankenfield

United States

Jeffrey Freeman

United States

Renata N Freitas

Brazil

Lara Friedlander

New Zealand

Elke Fröhlich-Reiterer

Austria

Klaus Frommer

Germany

Akio Fujimura

Japan

Birgit Fullerton

Germany

Katsuhiko Funai

United States

Martha M Funnell

United States

Clement E Furlong

United States

Monica M G Gabbay

Brazil

Avner Gal

Israel

Baptist Gallwitz

Germany

Madhur K Garg

India

Mark Geier

United States

Bizu Gelaye

United States

Lia Gentil

Canada

Daniela D.C.C.G. Gerardin

Brazil

Matthew Gilbert

United States

Bakary Adamu Girei

Nigeria

Fernando Giuffrida

Brazil

Nicole Glaser

United States

Francisco Gómez

Mexico

Marie-Paule Gonthier

France

Claudio Daniel Gonzalez

Argentine

Andrew Grandinetti

United States

Ignazio Grattagliano

Italy

Andrew Greenberg

United States

Randall Gretebeck

United States

Ryszard Grybos

Poland

Veronica Guarner

Mexico

Bruno Guigas

Holand

Anna Gumieniczek

Poland

Zelmira Guntsche

Argentine

Gerda-Maria Haas

Germany

Carlos Habitante

Brazil

Jorge M Haddad

Brazil

Stuart T Haines

United States

Serge Halimi

France

Alfredo Halpern

Brazil

Frederick Hamel

United States

Mohammad Hashemi

United States

Jun Hata

Japan

Noboru Hattori

Japan

Melvin Hayden

United States

Martina Heer

Germany

Lutz Heinemann

Germany

Helen H M Hermsdorff

Brazil

Emilio Herrera

Spain

Roman Herzig

Czech Republic

Denise Herzog

Canada

Maria Eliana Hidalgo

Chile

Andrew Hills

Australia

Tsutomu Hirano

Japan

Marcia Hiriart

Mexico

Frank Howarth

United Arab Emirates

Guan-Jhong Huang

Taiwan

Matthew Hulver

United States

Kuan-Yu Hung

Taiwan

Pamela Von Hurst

New Zealand

Chii-Min Hwu

Taiwan

Sung Ho Hyon

Argentine

Andrea Icks

Germany

Nobukazu Ishizaka

Japan

Faramarz I Ismail-Beigi

United States

Zafar H Israil

United States

Sushil K Jain

United States

Soong-Nang Jang

Korea

Judith Jansen

Germany

Leif Jansson

Sweden

Ellis Jensen

United States

Seul-Ki Jeong

Korea

Myung Ho Jeong

United States

Bo Ji

United States

David Jimenez-Pavon

Spain

Byung Jin Kim

Korea

Markandeya Jois

Australia

Hugh Jones

United Kingdom

Edmond K Kabagambe

United States

Sanjay Kalra

India

Nishan S Kalupahana

United States

Nashwa Nabil Kamal

India

Ippei Kanazawa

Japan

Kateřina Kaňková

Czech Republic

Güngör Karagüzel

Turkey

Katia P Karalis

Greece

Catherine Kargar

France

Brita Karlstrom

Sweden

Prabhdeep Kaur

India

Bisher Kawar

United Kingdom

Ryo Kawasaki

Australia

Patricia Kearney

Ireland

Roya Kelishadi

Iran

Wilma De Grava Kempinas

Brazil

Andre P Kengne

Australia

Romesh Khardori

United States

Madhu Khullar

India

Jan Kielstein

Germany

Takio Kitazawa

Japan

Jean Ko

United States

Rohit Kohli

United States

Marcia K Koike

Brazil

Alexandros Kokkinos

Greece

Peter Kokkinos

United States

Karel Kostev

Germany

Mario Kratz

United States

Karsten Kristiansen

Denmark

Anna Krook

Sweden

Chanda Kulkarni

India

Kavita Kumareswaran

United Kingdom

Avtar Lal

India

Philip J Lamey

United Kingdom

Rodrigo N Lamounier

Brazil

Zvi Laron

Israel

Márcio Lauria

Brazil

Albert Lecube

United States

Hong Kyu Lee

Korea

Warren Lee

Singapure

I-Te Lee

Taiwan

Ming-Che Lee

Taiwan

Yau-Jiunn Lee

United States

Paulo J M Leite

Brazil

Silmara Leite

Brazil

José Alexandre Curiacos De Almeida Leme

Brazil

Fezeu Leopold

France

Antonio Carlos Lerario

Brazil

Derek Leroith

United States

Hang Li

China

Tsai-Chung Li

Taiwan

Andreas Lindqvist

Sweden

Fábio Lira

Brazil

Jun-Li Liu

Canada

Mark Loftin

United States

Miguel Lopez

Spain

Oscar Lorenzo

Spain

Jun Lu

United States

Shou-En Lu

United States

Eliete El Luciano

Brazil

Manuel Luque-Ramirez

Spain

Tanica Lyngdoh

Switzerland

Ormond Macdougald

United States

Aristides Machado-Rodrigues

Portugal

Sten Madsbad

Denmark

Seiji Maeda

Japan

Winfried Maerz

Austria

Marcello Maggio

Italy

Maryam Maghsodi

Iran

Pamela Maher

United States

Koon Hou Mak

Korea

Brij M Makkar

India

Milos Maksimovic

Servia

Fani Ek Malerbi

Brazil

Raghunath Manchala

India

Carlos Mandarim-De-Lacerda

Brazil

Giulio Marchesini

Italy

Clarissa Martin

United Kingdom

Alberto Martinez

Spain

Danik Martirosyan

United States

Kazuko Masuo

Australia

Katia C Portero-Mclellan

Brazil

Wilson Luvizotto Medina

Brazil

Gema Medina-Gomez

Spain

Marco Andre Urbach Mezzasalma

Brazil

Ilias Migdalis

Greece

Adolpho Milech

Brazil

Marcio Miname

Brazil

Martin Miner

United States

Anoop Misra

India

Neal Mittman

United States

Ahmed Mohamadin

Egypt

Hicham Mohammadi

Maroc

Viswanathan Mohan

India

Marc L Molendijk

Holand

Antonella D'Arminio Monforte

Italy

Eduard Montanya

United States

Renan Magalhaes Montenegro-Jr

Brazil

Yong S K Moon

United States

Thomas Moore

United States

Samia Mora

United States

Rodrigo R M Moreira

Brazil

Katharine Morrison

United Kingdom

Michelle Mottola

Canada

Tomoatsu Mune

Japan

Ada Leticia B Murro

Brazil

Elza Muscelli

Italy

Iraj Nabipour

Iran

Mohamed Nagy

Egypt

Katsuyuki Nakajima

Japan

Takamitsu Nakamura

Japan

Jun Nakamura

Japan

Aline Nascimento

Brazil

Brandon M Nathan

United States

Carlos Antonio Negrato

Brazil

Azim Nejatizadeh

Iran

Chakib Nejjari

France

Sergio Neri

Italy

Markus Niessen

Switzerland

Nighat Nisar

Iran

Wester Eidi Nishimura

Brazil

Suneetha Nithyanandam

India

Fumio Nomura

Japan

Edoardo Dalla Nora

Italy

Victoria Novik

Chile

Andreas K Nussler

Germany

Luke B Nwoye

Saudi Arabia

Cheryl O'Malley

United States

Anthony O'Sullivan

Australia

Eiji Oda

Japan

Anthonia Ogbera

United Kingdom

Katashi Okoshi

Brazil

Erick De Oliveira

Brazil

Camila A Oliveira

Brazil

Claudia P M S Oliveira

Brazil

Jose Egidio Paulo De Oliveira

Brazil

Ann Olson

United States

Lisa Olsson

Sweden

Gamal A Omran

Egypt

Patricia Ostrosky

Mexico

Christoph Otto

Germany

Noriyuki Ouchi

Japan

Lila Oyama

Brazil

Maria Luiza M P Jorge

Brazil

Marco Paggi

Italy

Mariona Palou

Spain

Dimitrios Papamargaritis

United Kingdom

Nikolaos Papanas

Greece

Marcelo Papelbaum

Brazil

Candida R Parisi

Brazil

Marisa Passarelli

Brazil

Vinod Patel

United Kingdom

Hudsara Paula

Brazil

Michael Lynge Pedersen

Greenland

Ulrik Pedersen-Bjergaard

Denmark

Hermelinda Pedrosa

Brazil

Marina Pegoraro

Brazil

Sue Penckofer

United States

Xian'E Peng

China

Roger James Pepperell

Australia

Francisco A Pereira

Brazil

Maria Adelaide Albergaria Pereira

Brazil

Wolfgang Petrich

Germany

Claudia Pieper

Brazil

Gustavo Duarte Pimentel

Brazil

Somchai Pinlaor

Thailand

Juan Jose J Poderoso

Argentine

Maria Ponto

United Kingdom

Sergio Portal-Nunez

Spain

Victoria Vieira Potter

United States

Ally Prebtani

Canada

Luca Puccetti

Italy

Henry Punzi

United States

Saleem Saaed Qader

Sweden

Marcia Queiroz

Brazil

Denis Raccah

France

Roberto Raduan

Brazil

Laura Raimondi

Italy

Harpal S Randeva

United Kingdom

Athanasios Raptis

Greece

Karuna Rasineni

United States

Nelson N Rassi

Brazil

Rosangela R Réa

Brazil

Ellen Rearick

United States

André Fernandes Reis

Brazil

Eric Renard

France

Andrew Renehan

United Kingdom

Simone Renner

Germany

Geir Resaland

Norwegen

Rebecca M Reynolds

United Kingdom

Robespierre Ribeiro

Brazil

Raphael M Ritti-Dias

Brazil

Angela Albarosa Rivellese

Italy

Helena W Rodbard

United States

Amaia Rodríguez

Spain

Gustavo Rogatto

Brazil

Kendall Rogers

United States

Dora Romaguera

United Kingdom

Josefina Romaguera

United States

Hans Romijn

Holand

Gerard A Rongen

Holand

Robert S Rosenson

United States

Michael Roth

Switzerland

Gary Rothenberg

United States

Daniela Rubin

United States

Luis Ruilope

Spain

Jonatan Ruiz

Spain

Rovina Ruslami

Indonesia

Ingo Rustenbeck

Germany

Guy A Rutter

United Kingdom

Joao R Sa

Brazil

Omer Saatcioglu

Turkey

Anita A S Sachs

Brazil

Ikunosuke Sakurabayashi

Japan

Faouzi Saliba

France

Joao Eduardo J E N S Salles

Brazil

Stefanie Samietz

Germany

Mike Sampson

United Kingdom

Sergio Santoro

Brazil

Aruna Sarma

United States

Heike Sassmann

Germany

Kavithalakshmi Sataranatarajan

United States

Vincenzo Savica

Italy

Pikee Saxena

India

Richa Saxena

United States

Beatriz Dagord Schaan

Brazil

Mark A Schafer

United States

Helena Schmid

Brazil

Christian Schneider

Germany

Arthur Scholte

Holand

Sherwyn L Schwartz

United States

Laura Sciacca

Italy

Aidan Searle

United Kingdom

Antonio Secchi

Italy

Raymond Seet

Singapure

Adrianne Seiler

United States

Nihat Sen

Turkey

Muhittin A Serdar

Turkey

Stephen Setter

United States

Greg Seymour

New Zealand

Amy Shah

United States

Olfat Shaker

Egypt

Christian Sheline

United States

Wei Shen

United States

Min-Ho Shin

Korea

Zahra D Siadat

Iran

Dominique Sigaudot

France

Umberto Simeoni

France

Isaac Sinay

Argentine

Yuri Sinzato

Brazil

Nicos M Skordis

Cyprus

Vibe Skov

Denmark

Allan A Sneiderman

Canada

Alina Sokup

Poland

Bhavana Solanky

United Kingdom

Anna Solini

Italy

Yiqing Song

United States

Zhenyuan Song

United States

Jean Jorge Silva De Souza

Brazil

Lisa Stehno-Bittel

United States

Lars Christian Stene

Norwegen

Eliane E S Stevanato

Brazil

Rinke Stienstra

Holand

Corinne Stoop

Holand

Michael Strano

United States

Randi Streisand

United States

Maria L Strufaldi

Brazil

Junko Sugatani

Japan

Athula Sumathipala

United Kingdom

Sandra J Taler

United States

Peng Chiong Tan

Malaysia

Molly Tanenbaum

Iran

Shinichi Tanihara

Japan

Giovanni Targher

Italy

Olinda Tarzia

Brazil

Vladimir Tesar

Czech Republic

Aikaterini Theodoraki

Greece

David Thivel

France

David Thom

United States

Rebecca Thomson

Australia

Paul Thornalley

United Kingdom

Eduardo Tibirica

Brazil

Kulbhushan Tikoo

India

Peter Timmins

United Kingdom

Gaillard Trudy

United States

Apostolos Tsapas

Greece

Luiz A A Turatti

Brazil

Wojtek Tutak

United States

Konstantinos Tziomalos

Greece

Karin Ulrichs

Germany

Kausik Umanath

United States

Guillermo E Umpierrez

United States

Chioma Unachukwu

Nigeria

Ambika G Unnikrishnan

India

Denise M M Vancea

Brazil

Eszter Vanky

Norwegen

Maria Vanrompay

United States

Mireille Vasseur-Cognet

France

Frederick S Vaz

India

Jelena Vekic

Servia

Licio Velloso

Brazil

Sergio Vencio

Brazil

David L Vessely

United States

Marion L Vetter

United States

Loretta Vileikyte

United States

Jose Fernando Vilela-Martin

Brazil

Mohan Viswanathan

India

Gustavo Volpato

Brazil

Fabricio A Voltarelli

Brazil

Christina Voulgari

Greece

Patricia Vuguin

United States

Bernardo Leo Wajchenberg

Brazil

Pei-Wen Wang

Taiwan

Lijian Wang

United States

Christina Wassel

United States

Heike Weighardt

Germany

Gordon C Weir

United States

Karen L Whalen

United States

Ian Wilcox

Australia

Misty J Williams-Fritze

United States

Dane K Wukich

United States

Daozong Xia

China

Anny H Xiang

United States

Mohammad A Yakout

Egypt

Sho-Ichi Yamagishi

Japan

Kristina Yeghiazaryan

Germany

Deling Yin

United States

Kazuki Yokota

Japan

Koutaro Yokote

Japan

Hye Jin Yoo

Korea

Nasser Yousif

United States

Demin Yu

United States

Jong Won Yun

Korea

Shumei Yun

United States

Sato Yuzo

Japan

Claire Z Larter

Australia

Lenita Zajdenverg

Brazil

Maria Tereza Zanella

Brazil

Willibald Zeck

Austria

Hrvojka Marija Zeljko

Spain

Peixiang Zhang

United States

Zhi-Guang Zhou

China

Hong-Hao Zhou

Hong Kong

Giacomo Zoppini

Italy

